# HDAC Inhibition Induces Cell Cycle Arrest and Mesenchymal-Epithelial Transition in a Novel Pleural-Effusion Derived Uterine Carcinosarcoma Cell Line

**DOI:** 10.3389/pore.2021.636088

**Published:** 2021-03-26

**Authors:** Paul Stockhammer, Özlem Okumus, Luca Hegedus, Dominika Rittler, Till Ploenes, Thomas Herold, Stavros Kalbourtzis, Agnes Bankfalvi, Antje Sucker, Rainer Kimmig, Clemens Aigner, Balazs Hegedus

**Affiliations:** ^1^ Department of Thoracic Surgery, Ruhrlandklinik, West German Cancer Center, University Hospital Essen, University Duisburg-Essen, Essen, Germany; ^2^ Division of Thoracic Surgery, Medical University of Vienna, Vienna, Austria; ^3^ 2nd Institute of Pathology, Semmelweis University, Budapest, Hungary; ^4^ Institute of Pathology, University Hospital Essen, University Duisburg-Essen, Essen, Germany; ^5^ Department of Dermatology, University Hospital Essen, University Duisburg-Essen, Essen, Germany; ^6^ Department of Gynecology and Obstetrics, University Hospital Essen, University Duisburg-Essen, Essen, Germany

**Keywords:** uterine carcinosarcoma, targeted therapy, HDAC inhibition, epithelial-mesenchymal transition, mesenchymal-epithelial transition, ARID1A

## Abstract

**Objective:** Uterine carcinosarcoma (UCS) is a rare but highly aggressive malignancy with biphasic growth pattern. This morphology can be attributed to epithelial-mesenchymal transition (EMT) that often associates with tumor invasion and metastasis. Accordingly, we analyzed a novel patient-derived preclinical model to explore whether EMT is a potential target in UCS.

**Methods:** A novel UCS cell line (PF338) was established from the malignant pleural effusion of a 59-year-old patient at time of disease progression. Immunohistochemistry was performed in primary and metastatic tumor lesions. Oncogenic mutations were identified by next-generation sequencing. Viability assays and cell cycle analyses were used to test *in vitro* sensitivity to different standard and novel treatments. E-cadherin, β-catenin and pSMAD2 expressions were measured by immunoblot.

**Results:** Whereas immunohistochemistry of the metastatic tumor showed a predominantly sarcomatous vimentin positive tumor that has lost E-cadherin expression, PF338 cells demonstrated biphasic growth and carried mutations in *KRAS*, *PIK3CA*, *PTEN* and *ARID1A*. PF338 tumor cells were resistant to MEK- and TGF-β signaling-inhibition but sensitive to PIK3CA- and PARP-inhibition and first-line chemotherapeutics. Strikingly, histone deacetylase (HDAC) inhibition markedly reduced cell viability by inducing a dose-dependent G0/1 arrest and led to mesenchymal-epithelial transition as evidenced by morphological change and increased E-cadherin and β-catenin expression.

**Conclusions:** Our data suggest that HDAC inhibition is effective in a novel UCS cell line by interfering with both viability and differentiation. These findings emphasize the dynamic manner of EMT/MET and epigenetics and the importance of molecular profiling to pave the way for novel therapies in UCS.

## Introduction

Uterine carcinosarcoma (UCS) is a highly aggressive tumor that accounts for less than 5% of uterine malignancies [1, 2]. As a type of malignant mixed Müllerian tumors and related to poorly differentiated endometrial carcinomas, UCS is characterized by biphasic morphology with carcinomatous and sarcomatous differentiation [3, 4]. Compared to endometrial carcinoma, UCS has a worse prognosis with a high postoperative recurrence rate and a 5-year survival rate below 40% [5, 6]. In UCS, *TP53* has been identified as the most frequently mutated gene, followed by mutations in the PI3K pathway, *KRAS*, cell cycle regulators including *FBXW7* and chromatin remodeling and histone genes including *ARID1A* [7–9]. Importantly, UCS is the prototype tumor for epithelial-mesenchymal transition (EMT), a reversible biological process that associates with tumor progression and metastasis and in which epithelial cells transform into more invasive mesenchymal cells by losing their epithelial properties [10–12]. In UCS, several studies identified characteristic EMT-related expression signatures including active TGF-β signaling in tissue and cell lines [7, 11, 13, 14]. Interestingly, expression patterns of EMT-related markers including E-cadherin and ZEB1 were shown to differ between UCS carcinomatous and sarcomatous tumor areas [14]. The difference in E-cadherin expression is suggested to contribute to the biphasic growth pattern in UCS [15].

Recently, a transcriptome sequencing study in UCS demonstrated a strong correlation between EMT scores and epigenetic alterations [7]. In this regard, *ARID1A*, a commonly mutated chromatin remodeling gene in UCS, as well as the tumor suppressor *FBXW7* have been associated with EMT [16, 17]. Furthermore, mutations in either of them conferred sensitivity to histone deacetylase inhibition (HDACi) [18, 19]. In fact, histone modification by histone deacetylases is a major contributor to epigenetic changes in tumor cells and evidence suggests a functional role of HDAC inhibitors in EMT and phenotypic transformation of cancer cells [12, 20].

Suberoylanilide hydroxamic acid (SAHA), a pan-HDACi, and valproate are currently evaluated in various malignancies [20]. Although a significant portion of UCS harbor mutations in epigenetic regulators, evidence about HDACi in this entity is scarce. Previous studies found increased HDAC2 expression in endometrial stromal sarcomas and SAHA treatment in a uterine sarcoma cell line effectively suppressed growth [21, 22]. Accordingly, a recent study testing SAHA in UCS (NCT03509207) was initiated but soon after terminated due to issues in patient recruitment and access to medication. The potential of molecularly tailored therapies in UCS still needs to be further evaluated and novel UCS patient-derived cell lines are urgently needed as they are ideal models to study novel approaches. So far, there are just few reports of the successful establishment of UCS cell lines [23]. Accordingly, we aimed to investigate HDACi among other novel tailored approaches in a newly established UCS cell line. In this regard, we identified HDACi as a promising and reasonable approach targeting both epigenetics and EMT in UCS.

## Material and Methods

### Cell Culture and Reagents

The PF338 line was established from malignant pleural effusion. 5ml of effusion were centrifuged at 1,200 × *g* at room temperature for 10 min. The pellet was resuspended in RPMI1640 fortified by 10% FBS and 1% penicillin/streptomycin and seeded in a culture flask. More than 15 passages of the adherent cells with a minimum of three freezing-thawing cycles were done before experiments were initiated in order to use a tumor cell culture without non-tumorous cells. The A375 melanoma cell line was purchased from the ATCC and cultured in DMEM supplemented with 10% fetal bovine serum and 1% penicillin/streptomycin in culture flasks. Single Nucleotide Polymorphism (SNP) profiling was performed for PF338 and A375 tumor cell lines by Multiplex Cell Line Authentication (Multiplexion, Heidelberg, Germany) to confirm A375 cell line identity and PF338 unique cell line identity. Selumetinib, galunisertib, olaparib and BEZ235 were purchased from Selleck Chemicals (Houston, TX, United States) and dissolved in DMSO. SAHA and valproate were purchased from Sigma-Aldrich (St. Luis, MO, United States) and dissolved in DMSO and water, respectively. Paclitaxel (Kabi Fresenius, IL, United States) and cisplatin (Accord, Munich, Germany) were dissolved in 0.7% NaCl.

### Immunohistochemistry

Immunohistochemistry was performed using the Ventana BenchMark Ultra system (Roche Tissue Diagnostics, Grenzach-Vyhlen, Germany). 3 µm sections were prepared from formalin-fixed and paraffin embedded (FFPE) tumors and PF338 cellblock. The following primary antibodies were used: CD10 (Clone 56C6, 1:50, Leica Biosystems, Nussloch Germany), E-cadherin (Clone: NCH-38, Dako-Agilent, Waldbronn, Germany), vimentin (Clone: V9, Dako-Agilent, Waldbronn, Germany), progesterone receptor (Clone: 1E2; RTU, Roche Tissue Diagnostics) and estrogen receptor (Clone SP1, RTU, Roche Tissue Diagnostics). Color development was performed by the OptiView staining kit (Roche Tissue Diagnostics) followed by hematoxylin counterstaining. All stainings were evaluated by a senior pathologist (AB) and representative images were taken.

### Chemosensitivity Assays

Total protein amount-based Sulforhodamine B (SRB) assays were performed as follows. 5 × 10^3^ (PF338) or 2 × 10^3^ (A375) tumor cells /well were plated on the inner 60 wells of a 96-well plate and first incubated for 48 h. After 72 h of treatment with drugs, 10% TCA was used for fixation, followed by SRB dye (Sigma-Aldrich, St. Louis, MO, United States), and wash out with 1% acetic acid. 10mM Tris puffer dissolved the protein-bound dye and optical density (OD) was read at 570 nm by using a microplate reader (EL800, bioTec Instruments, Winooski, VT, United States). IC_50_ were calculated by using the CompuSyn software (ComboSyn, Inc., Paramus, NJ). Viability results are illustrated as ratio to control viability. For colony-formation assays, 1,000 tumor cells /well were plated on 6-well plates, incubated for 48 h and subsequently treated every 3–4 days with increasing drug concentrations for 10 days. 10% TCA was used for fixation, followed by SRB dye and wash out with 1% acetic acid. Colonies were counted manually. Experiments were repeated thrice.

### Cell Viability and Cell Cycle Analysis

In order to test the viability of cells after freezing and thawing at various passages the cell viability was measured on the NucleoCounter NC-3000TM system (Chemometec, Allerod, Denmark) using the cell viability reagents and protocol right after thawing and after 72 h in culture.

For cell cycle analysis, PF338 tumor cells were seeded on 6-well plates in 2 × 10^5^ cells/well concentration and incubated for 48 h, followed by 72 h of treatment. Cells were trypsinized and incubated with lysis buffer containing DAPI for 5 min at 37°C. Stabilization buffer was added, and cellular fluorescence was measured by the NucleoCounter NC-3000TM system (Chemometec, Allerod, Denmark). Cell cycle phases were identified based on the DNA content of the cells.

### Immunoblot

PF338 tumor cells were seeded into 6-well plates. After a recovery period of 24 h, cells were treated for 72 h with either HDACi (SAHA, valproate), galunisertib or solvent and precipitated with 6% TCA for 1 h, 4°C followed by centrifugation for 10 min at 9000 rpm. The total cellular protein pellets were resuspended in electrophoresis sample buffer (62.5 mM Tris–HCl, pH 6.8, 2% SDS, 10% glycerol, 5 mM EDTA, 125 mg/ml urea, 100 mM dithiothreitol) to be later loaded on 10% acrylamide gels in equal protein amounts. For immunostaining rabbit anti-E-cadherin (Cell Signaling, 24E10, 1:1,000), anti-beta-catenin (Santa Cruz, Sc-7199, 1:500), anti-pSMAD2 (Cell Signaling, 138D4, 1:1,000) and polyclonal anti-beta-tubulin (Abcam, ab6046, 1:1,000) were used. As secondary antibody HRP-conjugated anti-rabbit antibody (Jackson ImmunoResearch, 1:10.000) was used. For development ECL Western Blotting Substrate (Thermo Scientific, Waltham, MS, United States) was applied followed by luminography. Three independent experiments were performed.

### Next-Generation Sequencing (NGS)

DNA from PF338 cells was isolated according to the manual’s instructions by using DNeasy Blood and Tissue Kit (Qiagen, MD, United States). FFPE tissue DNA was isolated according to the manual’s instructions by using QIAamp DNA FFPE Tissue Kit (Qiagen, MD, United States). DNA concentrations were determined by Qubit^®^ 2.0 Fluorometer dsDNA HS assay kit (LifeTechnologies, CA, United States).

A total amount of 45 ng DNA was used for multiplex-PCR. Multiplex PCR and purification were performed with the GeneRead DNAseq Custom Panel and PCR Kit V2 (Qiagen, MD, United States) and Agencourt^®^ AMPure^®^ XP Beads (Beckman, CA, United States). The library preparation was performed with NEBNext Ultra DNA Library Prep Set for Illumina (New England Biolabs, MA, United States), according to the manufacturer’s recommendations by using 24 different indices per run. The pooled library was sequenced on MiSeq (Illumina; 2 × 150 bases paired-end run) and analyzed by Biomedical Genomics Workbench (CLC Bio, Qiagen, MD, United States). For targeted sequencing a customized comprehensive cancer-panel was designed containing regions of interest.

### Time-Lapse Video Microscopy

Video microscopy was performed as previously [24] and now described in Supplementary materials.

### Statistics

Two-way ANOVA with Bonferroni posttest was applied to describe significant differences between cell lines and treatment lines. One-way ANOVA with Dunn´s multiple comparison test was applied to identify significant differences between treatment lines. *p < 0.05; **p < 0.01; ***p < 0.001, and ****p < 0.0001 represented significant differences. All calculations were done in GraphPad Prism 8 (GraphPad Software Inc., San Diego, CA).

## Results

### Clinical History

**FIGURE 1:**
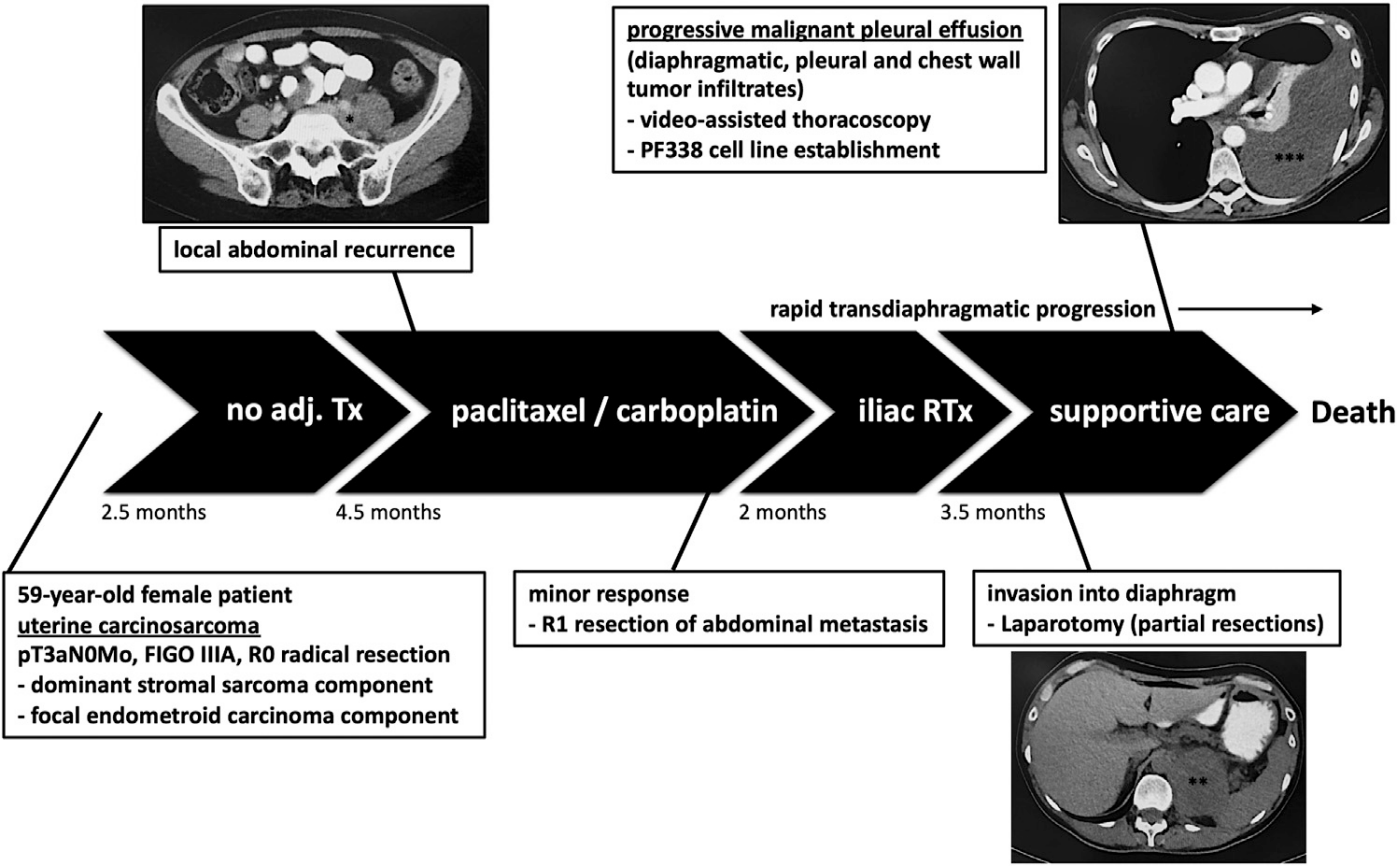
Patient’s history. The patient underwent resection without adjuvant treatment (Tx). Due to retroperitoneal recurrence, chemotherapy was initiated followed by surgery and adjuvant radiation therapy (RTx). However, the tumor progressed leading to pleural effusion justifying laparotomy and video-assisted thoracoscopy. At that time we established the pleural-effusion derived tumor cell line PF338. *retroperitoneal recurrence; **tumor infiltrate; ***transdiaphragmatic spread and pleural effusion.

A 59-year-old female patient was diagnosed with UCS and underwent radical resection yielding a pT3aN0M0 FIGO IIIA tumor containing both a dominant stromal sarcomatous and a focal endometroid carcinomatous component (Figure 1). No adjuvant treatment was applied, however, 2.5 months later the patient developed retroperitoneal recurrence, for which chemotherapy consisting of three cycles paclitaxel/carboplatin was started. Re-staging indicated a tumor response and thus three additional cycles of chemotherapy were applied, followed by resection of the metastatic lesion. Histological analyses at that time revealed positive tumor margins, justifying adjuvant iliac radiation therapy. Due to rapid locoregional spread infiltrating diaphragm, chest wall and pleura accompanied by accumulating pleural effusions, the patient underwent partial resections including laparotomy and video-assisted thoracoscopy. Finally, treatment was switched to supportive chemotherapy, however, the patient continued to deteriorate and succumbed to the disease 12.5 months after initial diagnosis.

### Histological Tumor Characterization

**FIGURE 2:**
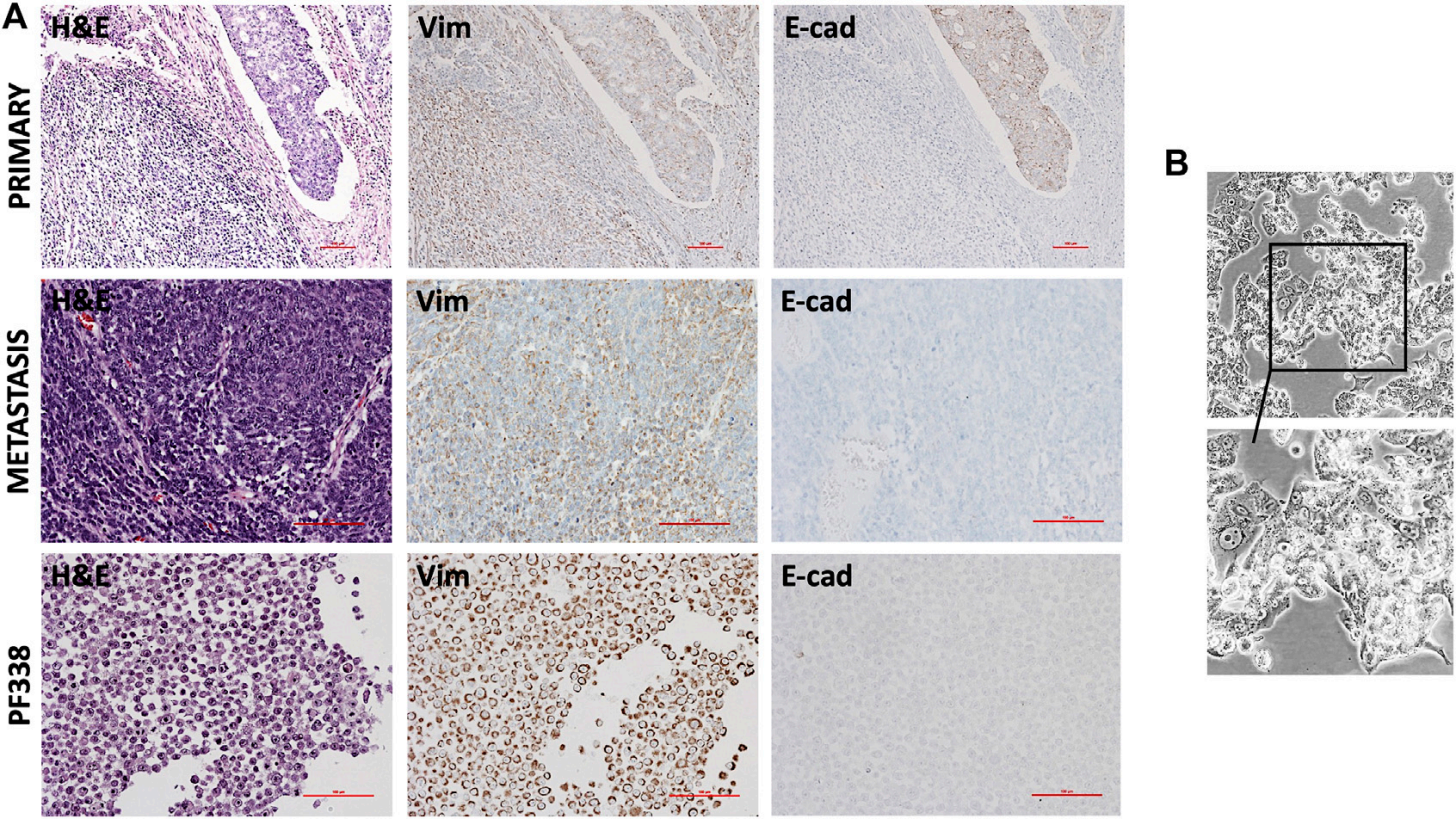
Histopathological and *in vitro* characterization **(A)** Hematoxylin and eosin (H & E) and immunohistochemical stainings of the primary tumor, the recurrent tumor and of PF338 tumor cells. Whereas the primary tumor expressed both E-cadherin and vimentin, E-cadherin expression was lost in both the metastatic lesion and the cell line **(B)** PF338 tumor cell growth *in vitro*.

To compare the primary tumor lesion at diagnosis with the metastatic tumor we performed immunohistochemical analyses (Figure 2A). At diagnosis, the tumor contained a dominant sarcomatous component positive for vimentin and a focal carcinomatous component positive for E-cadherin. At recurrence, hematoxylin and eosin (H&E) staining revealed mainly spindle-shaped tumor cells diffusely positive for vimentin but negative for E-cadherin. To confirm the origin of the metastatic tumor, we demonstrated that tumor cells were, although only in foci, positive for CD10, a marker for Müllerian system-derived neoplastic mesenchymal cells [25] (Supplementary Figure S1A). In addition, a proliferation rate of up to 60% was detected in hotspot areas by Ki67 staining (Supplementary Figure S1A). Stainings for estrogen- and progesterone-receptor (ER, PR) indicated only heterogeneous nuclear expression patterns (Supplementary Figure S1A).

Taken together, the metastatic resected specimen was highly proliferative, had lost the carcinomatous histological component and was heterogeneously positive for CD10, ER and PR.

### PF338 Cell Line Establishment and Mutational Characterization

At time of progression, we obtained pleural effusion and could successfully establish the PF338 UCS cell line. Congruent to the metastatic tissue, immunohistochemical stainings of the cell block indicated focal positivity for CD10, strong positivity for vimentin and negative staining for E-cadherin (Figure 2A; Supplementary Figure S1B). *In vitro*, PF338 tumor cells demonstrated a biphasic growth pattern consisting of an epithelial-like component growing in a monolayer and a mesenchymal-like component growing in multiple layers (Figure 2B; Supplementary Video S1). In order to study the effect of multiple freezing cycles on tumor cell viability, we compared viability of PF338 cell passage 16 vs. passage 26 and found no differences (viability right after thawing 81.6 vs. 84.2%, viability after 72 h in culture 98.6% vs. 97.2%).

To illuminate the mutational background of the tumor cell line and the primary/metastatic tumor tissues we performed NGS for a predefined mutational panel that included the most commonly mutated genes in UCS (Supplementary Table S1). We identified a G13C mutation in *KRAS*, a R130Q mutation in *PTEN* and a mutation in *ARID1A*. Interestingly, an R93Q mutation in *PIK3CA* was only detected in the metastatic tumor and the cell line but not in the primary tumor.

### PF338 Tumor Cells Are Sensitive to Cisplatin and Paclitaxel *in vitro*

**FIGURE 3:**
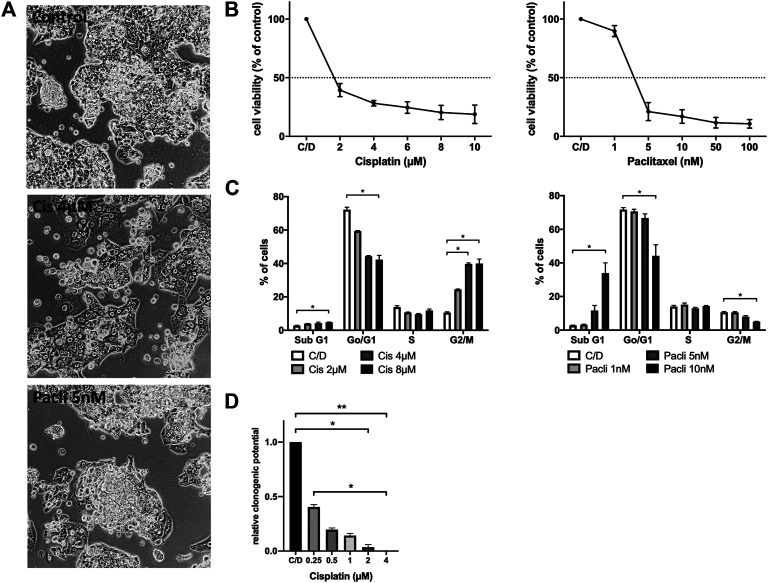
PF338 tumor cells are sensitive to both cisplatin and paclitaxel **(A)** Cisplatin reduced the mesenchymal-like cell fraction of PF338 cells **(B)** Cells were sensitive to cisplatin (IC_50_: 0.96 µM) and paclitaxel (IC_50_: 3.81 nM) **(C)** Cisplatin induced G2/M arrest and paclitaxel apoptosis **(D)** Tumor cells could still form colonies when treated with IC_50_ concentrations of cisplatin. Error bars = means ± SE from three repeats. C/D, control, *p < 0.05, **p < 0.01.

In order to test whether PF338 cells are sensitive to standard-of-care UCS treatment paclitaxel plus platinum-based chemotherapy we performed *in vitro* sensitivity testing for both drugs. Accordingly, PF338 cells were sensitive to cisplatin and paclitaxel, with IC_50_ values of 0.96 µM and 3.81 nM, respectively (Figures 3A,B). Interestingly, whereas cisplatin induced morphology changes to a more uniform, flat phenotype, paclitaxel treatment did not interfere with morphology (Figure 3A). Cell cycle analyses for cisplatin revealed a dose-dependent G2/M arrest, whereas paclitaxel induced apoptosis in a dose-dependent manner (Figure 3C). Despite being sensitive to cisplatin, a significant fraction of cells (15%) remained viable when treated with cisplatin at IC_50_ concentrations for 10 days (Figure 3D; Supplementary Figure S2).

### Targeted Therapy With Kinase Inhibitors in PF338 Tumor Cells

**FIGURE 4:**
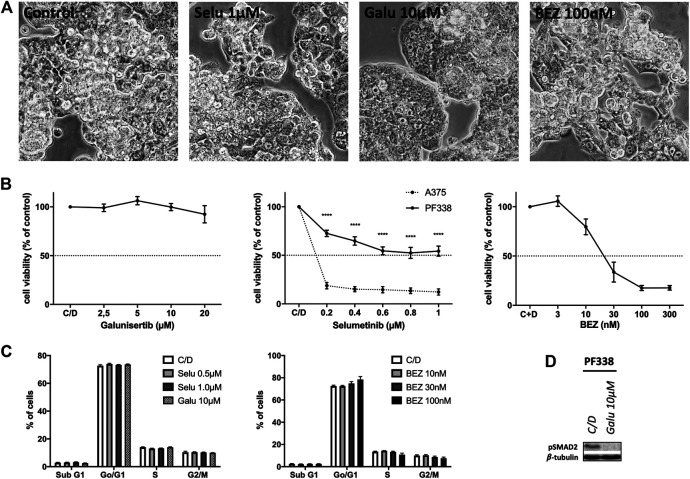
PF338 tumor cells are resistant to MEK and TGF-βRI-kinase inhibition but sensitive to PI3K inhibition **(A)** selumetinib (Selu), galunisertib (Galu) and BEZ235 (BEZ) did not affect PF338 cell morphology **(B)** PF338 cells were resistant to selumetinib (A375 cells were used as control) and galunisertib, but sensitive to BEZ235 (IC_50_: 63.42 nM) **(C)** BEZ235 induced a modest G0/G1 arrest **(D)** Galunisertib reduced pSMAD2 expression. Error bars = means ± SE from three repeats. C/D, control, ****p < 0.0001.

In order to test whether *KRAS*-mutant PF338 cells are sensitive to MAPK pathway inhibition, we used selumetinib, a MEK inhibitor that has been tested in *KRAS-*mutant tumors [26]. As sensitive control we used the *BRAFV600E* mutant A375 melanoma line [27]. We also tested the dual PI3K/mTOR inhibitor BEZ235 due to the mutations in the PI3K pathway and also treated the cells with galunisertib, a TGF-βRI-kinase inhibitor. PF338 cells were resistant to both selumetinib and galunisertib but strongly sensitive to PI3K/mTOR inhibition (IC_50_: 63.42 nM) (Figures 4A,B). Cell cycle analyses revealed no changes upon selumetinib or galunisertib treatment and only a modest dose-dependent G0/G1 arrest upon BEZ235 treatment (Figure 4C). However, despite the resistance to galunisertib, pSMAD2 expression was abrogated by the treatment (Figure 4D).

### PF338 Tumor Cells Are Highly Sensitive to HDAC and PARP Inhibition

**FIGURE 5:**
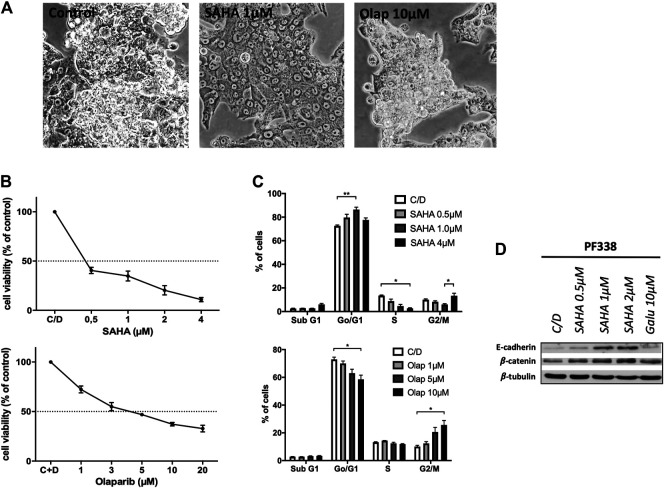
PF338 tumor cells are highly sensitive to HDAC inhibition and change differentiation **(A)** SAHA treatment changed morphology from biphasic to epithelial **(B)** Cells were sensitive to SAHA (IC_50_: 0.38 µM) and olaparib (IC_50_: 4.60 µM) **(C)** SAHA induced a G0/1 arrest and with higher concentrations both a G2M arrest and apoptosis; olaparib induced G2/M arrest **(D)** SAHA dose-dependently upregulated the expression of E-cadherin and β-catenin. Error bars = means ± SE from three repeats. C/D, control, *p < 0.05, **p < 0.01.

Recent work in ovarian cancer demonstrated that mutations in *ARID1A* confer sensitivity to HDACi [18]. Furthermore, UCS frequently harbor alterations in cell cycle regulators and thus may show susceptibility to certain targeted therapies including PARP inhibitors [7]. Strikingly, PF338 cells were sensitive to both HDACi SAHA and PARP inhibitor olaparib with IC_50_ of 0.38 and 4.60 µM, respectively (Figures 5A,B). SAHA treatment induced two distinct cell cycle patterns: a dose dependent G0/1 arrest at low drug concentrations and both a G2/M arrest and induction of apoptosis at high concentrations. In contrary, olaparib-treated cells went into G2/M arrest in a dose-dependent manner (Figure 5C). Importantly, PF338 tumor cells changed morphology from the initial biphasic to an epithelial phenotype upon SAHA treatment (Figure 5A; Supplementary Video S2). These phenotypic changes were accompanied by a dose-dependent upregulation of epithelial markers E-cadherin and—to a lesser extent—β-catenin (Figure 5D). Importantly, phenotypic and expression changes were also observed upon treatment with high-dose SAHA (4 µM) or valproate (Supplementary Figures S3A–D).

## Discussion

The highly invasive and aggressive growth pattern of UCS in combination with a still poorly understood tumorigenic molecular background contribute to dismal patient prognosis [28]. Due to the low incidence and a very limited number of clinical trials, multicenter studies investigating novel agents and combinations are of utmost importance to offer evidence-based therapies. Accordingly, patient-derived tumor cell lines are crucial to identify novel therapeutics. Our study describes a newly established UCS cell line and to our best knowledge is the first report of *in vitro* HDAC inhibition in UCS.

Our patient underwent multiple surgeries followed by chemo- and radiotherapy; however, despite initial treatment response she rapidly relapsed and succumbed to the disease. There is still no consensus regarding the optimal therapeutic management for UCS patients and as in our case, UCS tumors tend to relapse within two years after diagnosis despite initial systemic treatment response [29]. Although certain multimodal approaches were shown to be potentially effective in UCS patients, prospective validation studies and novel approaches are urgently needed [30].

In our case, the resected metastatic lesion showed mainly sarcomatous differentiated tumor cells without E-cadherin expression. In contrast, a dominant sarcomatous and a focal carcinomatous E-cadherin positive histological component were present at initial diagnosis. This histological change together with rapid progression is in line with a recent study identifying sarcomatous component on recurrence to be significantly associated with poor disease-free interval [31]. Metastatic UCS lesions were described as predominantly carcinomatous or biphasic tumors but no pure sarcomas in a large retrospective cohort [1]. To study the dynamic process of differentiation changes in UCS, a biphasic cell model representing both morphologies *in vitro* is an invaluable asset. However, there are just a few patient-derived UCS cell lines, and the majority of those are of sarcomatous differentiation [23]. Importantly, our novel UCS cell line demonstrated biphasic differentiation as illustrated by *in vitro* morphology and growth. Of note, a similar biphasic phenotype *in vitro* was described for UCS cell lines SNU-685 and EMTOKA [32, 33].

For the majority of previously established UCS lines analyses of driver mutations were not performed. PF338 cells harbor the G13C mutation in *KRAS* but are resistant against MEK-inhibition. According to the fourth dataset of the AACR GENIE project, the majority of *KRAS* ^*G13C*^ mutant tumors are non-small cell lung cancers and colorectal carcinomas. However, four of the *KRAS* ^*G13C*^ mutant tumors were uterine cancers [34]. To the best of our best knowledge, there is no data available regarding RAS/MAPK pathway inhibition in *KRAS* ^*G13C*^ cells and according to the ATCC there are only two lung adenocarcinoma lines with this mutation. The other *KRAS* mutant UCS line TU-ECS-1has the more common G12D mutation in addition to several *TP53* mutations [35].

PF338 tumor cells harbored mutations in *PIK3CA* and *PTEN* and were sensitive to dual PI3K/mTOR inhibition. Importantly, alterations in the PI3K pathway have been described for the majority of UCS and about one quarter of UCS demonstrate simultaneous mutations in *PTEN* and *PIK3CA* [7–9]. Several clinical trials are investigating PI3K pathway inhibition in endometrial carcinoma but data for UCS are missing [7]. Interestingly, in our case, the *PIK3CA* mutation was only present at the time of metastasis and not at diagnosis. Similar findings were reported in a melanoma study in which one of eight tested cases had a *PIK3CA* mutation only present in the metastatic lesion [36]. However, in UCS, McConechy et al. found that *PIK3CA* mutations were uniformly present in both the diagnostic and metastatic lesions and hence they hypothesized that such mutations may occur early during tumorigenesis [9].

As in our case, in patients with recurrent UCS, chemotherapy consisting of different combinations of carboplatin/cisplatin, paclitaxel and ifosfamide are treatment of choice [4–6]. Our patient initially responded to chemotherapy and in line with that, PF338 cells were sensitive to both cisplatin and paclitaxel *in vitro*. These findings are similar to the data from the TU-ESC-1 cell line which was shown to be sensitive to both drugs as well [35]. However, our long-term treatment showed that a fraction of PF338 cells remained viable when treated with high cisplatin concentrations. This could explain why UCS patients tend to respond to chemotherapy at first but ultimately relapse within two years [29].

Due to emerging evidence suggesting a functional role of EMT in UCS tumorigenesis and its biphasic growth by definition, UCS is the prototype tumor to study EMT [4, 11]. Importantly, EMT has been linked to the transition from endometrial carcinoma to carcinosarcoma and to the metastatic process during disease progression [37]. In two sarcomatoid UCS lines certain TGF-β family members were found to be expressed and inhibition with galunisertib could partially abrogate TGF-β mediated effects on proliferation, migration and EMT. Importantly, galunisertib alone did reduce pSMAD2 expression but did not affect cell viability [13]. This is in line with our data of no change in cell viability, cell cycle distribution and cell morphology but a downregulation of pSMAD2 expression following galunisertib treatment. Putting this into context, blocking TGF-β signaling in UCS might not be effective as single agent but rather in combinatory approaches. Accordingly, Dwivedi et al. could recently demonstrate promising results by combining galunisertib with standard chemotherapy *in vivo* by treating xenografts established from a UCS cell line with high relative TGF-β and TGF-βRI expression [38]. A phase IB trial investigating galunisertib with chemotherapy in UCS is currently recruiting patients (NCT03206177). Given that our PF338 tumor cells did not change viability or morphology to TGF-β signaling inhibition despite reduced pSMAD2 expression upon galunisertib treatment, we concluded that TGF-β signaling may not be the major driver for EMT and cell proliferation in our model. Additional investigations will be necessary to better describe the role of TGF-β blockade in UCS.

The PARP inhibitor olaparib affected viability by inducing a G2/M arrest in PF338 cells. Alterations in cell cycle regulators, which are frequently detected in UCS, potentially induce susceptibility to PARP inhibition [7]. Furthermore, a large study investigating PARP1 expression in various tumors found that the majority of UCS markedly overexpressed PARP1 [39]. However, data with regard to FDA-approved PARP inhibitors in UCS are missing and our data is the first to suggest PARP inhibition as effective in UCS.

We detected an *ARID1A* mutation in our case. Importantly, 10–30% of UCS harbor mutations in *ARID1A*, representing the most frequently altered chromatin remodeling gene in UCS [7–9]. Recent evidence demonstrated a link between EMT and epigenetic alterations in UCS [7]. One study reported that loss of *ARID1A* leads to the expression of EMT genes and epithelial transdifferentiation in the endometrium [16]. Furthermore, ARID1A normally suppresses certain HDACs and tumor cells with *ARID1A* mutations lose this feedback, become HDAC dependent and hence highly sensitive to HDACi [18, 40]. Our findings that SAHA and valproate interfered with cell viability, cell cycle distribution and cell differentiation in ARID1A mutant PF338 tumor cells strongly support this hypothesis. Upon SAHA treatment, tumor cells dose-dependently underwent reverse EMT, a process also called mesenchymal-epithelial transition (MET), characterized by increased E-cadherin and β-catenin expression and morphological re-differentiation into an epithelial phenotype. To the best of our knowledge, the current study provides the first evidence showing HDACi to be effective in UCS *in vitro* by interfering with EMT/MET. In fact, re-expression of E-cadherin is considered a major marker of MET in tumor cells [41]. Our findings of HDACi interfering with EMT/MET are in line with a number of studies in different malignancies [12]. Downregulation of E-cadherin by epigenetic changes in cancer has been extensively described and linked to tumor invasiveness, dissemination and progression [42]. In contrast, MET, the reverse process, has been linked to tumor cell re-differentiation [43]. Considering the fact that the majority of UCS tumors relapse after radical surgery, targeting histone modification and differentiation by HDACi might be an effective novel approach to prevent UCS tumor cells from metastasizing. Although a recent clinical trial (NCT03509207) already aimed to investigate SAHA in UCS, molecular explanations justifying its rational and clinical implementation were scarce and limited to a study in a uterine sarcoma cell line [21, 22]. HDAC inhibition is a rapidly growing field in cancer therapy throughout various malignancies. Based on our findings, targeting epigenetics and consequently EMT by using HDACi in UCS might be a promising novel approach and should be further explored in future clinical trials. Furthermore, identifying the mutational background of UCS at time of tumor progression is of utmost importance to better predict sensitivity to targeted therapies including PI3K pathway, PARP and HDAC inhibition.

## Data Availability

The original contributions presented in the study are included in the article/Supplementary Material, further inquiries can be directed to the corresponding author.
