# Supplementary material for: HDAC Inhibition Induces Cell Cycle Arrest and Mesenchymal-Epithelial Transition in a Novel Pleural-Effusion Derived Uterine Carcinosarcoma Cell Line
Source: Pathol Oncol Res. 2021 Mar 26;27:636088. doi: 10.3389/pore.2021.636088 (PMC8262245; doi:10.3389/pore.2021.636088)
Supplement: Supplementary file 1 [file DataSheet1.DOCX]

**Supplementary materials**

**Figure legends:**

**Figure S1. Immunohistochemical markers of uterine tumors**

**(A)** Immunohistochemistry in the metastatic lesion showing focal positivity for CD10 and modest nuclear positivity for both estrogen- (ER) and progesterone-receptor (PR). The metastatic lesion stained strongly positive for Ki67 of up to 60% in hotspot areas. **(B)** In PF338 tumor cells, there was a focal positivity for marker CD10.

**Figure S2. Long-term treatment with cisplatin**

Long-term treatment for 10 days with cisplatin was performed. Accordingly, PF338 tumor cells were able to form reasonable colonies (15% of control) up to the cisplatin treatment concentrations of 1µM.

**Figure S3. The effect of high-dose SAHA and valproate on PF338 tumor cells**

**(A)** A change in PF338 tumor cell morphology to a more epithelial phenotype was seen after treatment with 2mM valproate and when treated with high-dose (2µM or 4µM) of SAHA. **(B)** PF338 tumor cells were sensitive to valproate (IC_50_ of 2.44mM) and **(C)** valproate upregulated both E-cadherin and β-catenin expression upon treatment. **(D)** Also, high-doses of SAHA (2µM and 4µM) strongly upregulated E-cadherin expression. Error bars demonstrate means ±SE from three independent experimental repeats. C/D, control; Val, valproate.

**Supplementary movie 1. PF338 in vitro growth illustrated by 2D video microscopy**

Cells were plated and cultured in 24-well plates with DMEM medium +10% FCS. Subsequently, a CO_2_-independent medium (Invitrogen, CA, USA) +10% FCS and 4mM glutamine was used and PF338 tumor cells were kept in a custom-designed incubator constructed around an inverted phase-contrast microscope (World Precision Instruments, FL, USA). Images were taken every 5min from 3 neighboring microscopic fields for 48 hours.

**Supplementary movie 2. PF338 in vitro growth under SAHA treatment illustrated by 2D video microscopy**

Cells were plated and cultured in 24-well plates with DMEM medium +10% FCS. Subsequently, a CO_2_-independent medium (Invitrogen, CA, USA) +10% FCS and 4mM glutamine was used and PF338 tumor cells were kept in a custom-designed incubator constructed around an inverted phase-contrast microscope (World Precision Instruments, FL, USA). Treatment was started after 24 hours with either SAHA or solvent. Images were taken every 5min from 3 neighboring microscopic fields for 24 hours prior to treatment and 48 hours after treatment.

**Supplementary table legends**

**SUPPLEMENTARY TABLE 1. Next-Generation sequencing (NGS) panel for mutational analyses of tumor tissues and PF338 tumor cells**

| **Gene** | **Primary tumor** | **Metastatic tumor** | **PF338 cell line** |
| --- | --- | --- | --- |
| KRAS | c.37G>T (G13C) | c.37G>T (G13C) | c.37G>T (G13C) |
| PIK3CA | wt | c.278G>A (R93Q) | c.278G>A (R93Q) |
| PTEN | c.389G>A (R130Q) | c.389G>A (R130Q) | c.389G>A (R130Q) |
| ARID1A | P1326fs*155 | P1326fs*155 | P1326fs*155 |
| FBXW7 | wt | wt | wt |
| TP53 | wt | wt | wt |
| MET | wt | wt | wt |
| NRAS | wt | wt | wt |
| RET | wt | wt | wt |
| BRAF | wt | wt | wt |
| EGFR | wt | wt | wt |
| ERBB2 | wt | wt | wt |
| FGFR1 | wt | wt | wt |
| FGFR3 | wt | wt | wt |
| HRAS | wt | wt | wt |
| IDH1 | wt | wt | wt |
| IDH2 | wt | wt | wt |
| KIT | wt | wt | wt |
| PDGFRα | wt | wt | wt |
| STK11 | wt | wt | wt |
| TERT promoter | wt | wt | wt |
