# Supplementary figures and images for: HDAC Inhibition Induces Cell Cycle Arrest and Mesenchymal-Epithelial Transition in a Novel Pleural-Effusion Derived Uterine Carcinosarcoma Cell Line
Source: Pathol Oncol Res. 2021 Mar 26;27:636088. doi: 10.3389/pore.2021.636088 (PMC8262245; doi:10.3389/pore.2021.636088)

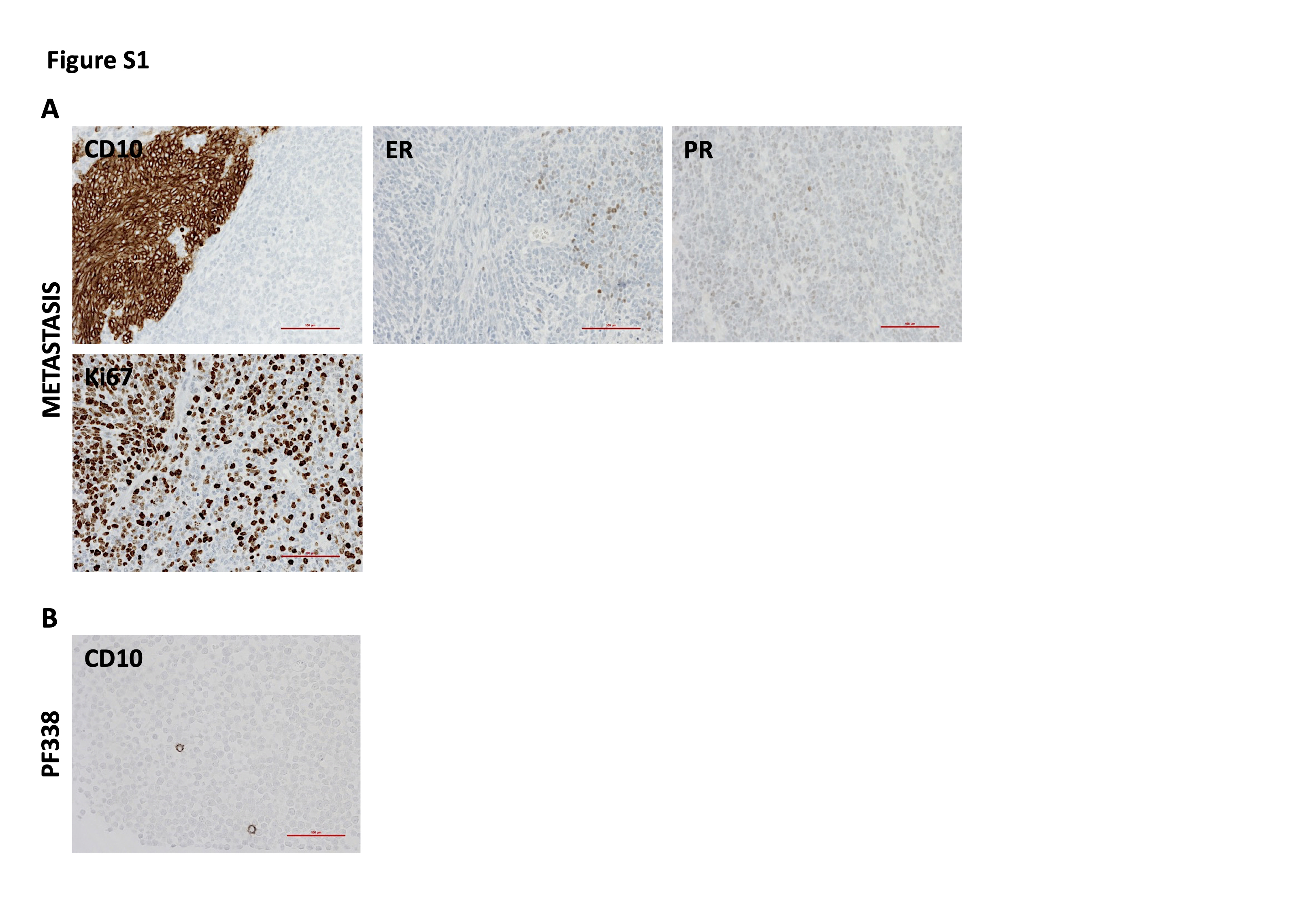

Supplement: Supplementary file 2 [file Image1.JPEG]

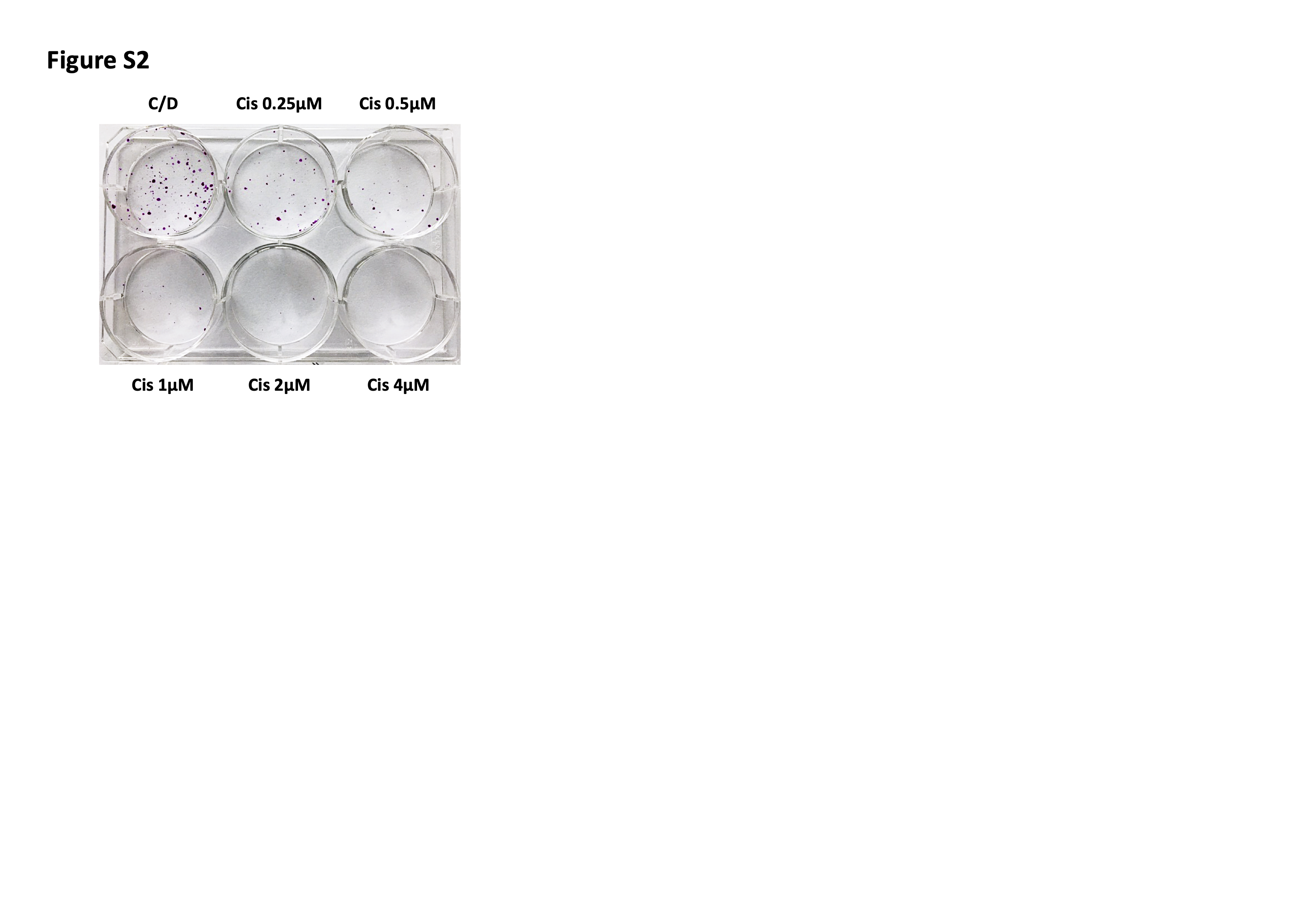

Supplement: Supplementary file 3 [file Image2.JPEG]

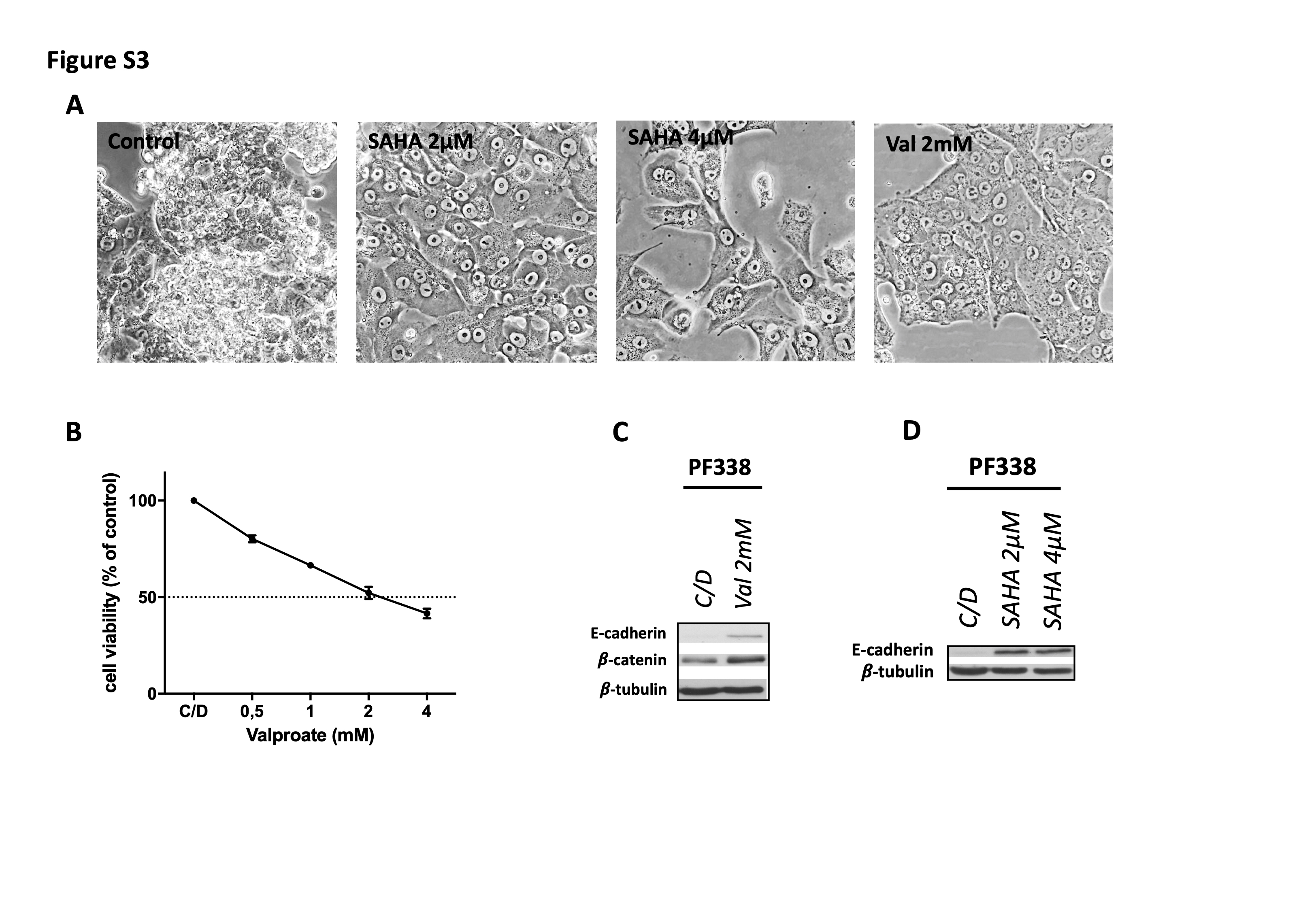

Supplement: Supplementary file 4 [file Image3.JPEG]
